# Strukturierte Befundung in der Radiologie – Chance für die radiologische Jugend?

**DOI:** 10.1007/s00117-021-00826-2

**Published:** 2021-03-19

**Authors:** Benjamin Sigl, Christian Herold

**Affiliations:** Universitätsklinik für Radiologie und Nuklearmedizin, Medizinische Universität Wien, 1090 Wien, Österreich

**Darum geht es:**

✓: Vor- und Nachteile strukturierter Befundung
✓: Etablierte Praxisfelder strukturierter Befundung
✓: Quellen für Befundvorlagen und standardisiertes Vokabular

Mit dem technischen Fortschritt in der Radiologie wachsen auch ihre Einsatzgebiete. Die Fragestellungen werden vielfältiger, der Befundumsatz steigt und die Arbeitsbelastung der Radiologen nimmt zu. Der wachsenden Nachfrage kann jedoch selten auch der Stellenschlüssel in den radiologischen Abteilungen und Praxen adäquat angepasst werden. Auch der radiologische Nachwuchs kämpft vielerorts mit dem Problem dieser Arbeitsverdichtung. Als Teil einer möglichen Lösung wird hierbei häufig die strukturierte Befundung vorgeschlagen. Doch warum hat sich dieses „Wundermittel“ der Befunderstellung nicht schon flächendeckend durchgesetzt, obwohl es seit Jahrzehnten bekannt ist? Schließlich steigen Publikationen zu diesem Thema bereits seit der Jahrtausendwende exponentiell an. Was versteht man unter strukturierter Befundung genau? Welche Vorlagen werden bereits verwendet und welche Varianten sind auf dem Vormarsch? Was verhindert bisher einen Siegeszug der strukturierten Befundung? Und welche Chancen bietet sie gerade für die radiologische Jugend?

Der Begriff „Strukturierte Befundung“ enthält in der Radiologie ein breites Spektrum in Bezug auf den Grad dieser Strukturierung: Letztendlich gilt bereits jeder Befundbericht als strukturiert, der im Aufbau die DIN 6827‑5 erfüllt. Nach dieser Norm werden Befunde in die Abschnitte „Angaben zum Patienten“, „Angaben im Rahmen der Röntgenverordnung“ (optional), „Angaben zur Untersuchung“, „medizinischer Inhalt“ und „Angaben zum Befundbericht“ gegliedert [1]. Dieser Mindestanforderung dürften die allermeisten Befunde bereits entsprechen; sie bilden die erste Stufe der strukturierten Befundung. Werden zusätzlich vorformulierte Textbausteine verwendet, sind die Anforderungen der zweiten Stufe erfüllt. Die dritte Stufe wird erreicht, wenn diese Textbausteine allein aus kontrollierten Vokabularien bestehen, beispielsweise dem BI-RADS-Lexikon [2]. Im Weiteren soll das Augenmerk auf Befunde höherer Strukturierungsgrade, also der Stufe 2 und 3 gelegt werden.

Es wird bereits ein gewisses Zeiteinsparpotenzial ersichtlich, wenn Formulierungen vorgegeben sind und technische Daten, wie MR-Sequenzen oder CT-Protokolle, automatisch eingefügt werden können. Durch den gleichförmigen Aufbau der Befunde wird eine schnellere Orientierung sowohl für Radiologen als auch Zuweiser ermöglicht, sodass dieses Potenzial weiter ausgeschöpft werden kann. Betrachtet man diesen Punkt etwas genauer, wird neben dem zeitlichen Aspekt auch ein weiterer wesentlicher Vorteil der strukturierten Befundung deutlich: Die Genauigkeit der Sprache nimmt zu [3]. Damit kann das interdisziplinäre und überregionale Verständnis deutlich gefördert [4] und letztlich die Patientenversorgung verbessert werden [5]. So können beispielsweise lokal übliche Sprachwendungen oder Abkürzung in anderen Regionen für Unklarheiten sorgen. Kein Wunder also, dass in der Qualitätssicherung die Vereinheitlichung von Befunden einen Schwerpunkt bildet, mit dem sich viele Fachgesellschaften auseinandersetzen [6], zum Beispiel bei den schon länger etablierten Planungs-CTs im Rahmen einer TAVI („transcatheter aortic-valve implantation“; [7]). Eine einheitliche Terminologie bringt nicht nur im deutschsprachigen Raum Klarheit. Gerade in Zeiten der innereuropäischen Öffnung gewinnt auch die grenzüberschreitende Medizin an Relevanz – und mit ihr die korrekte Übersetzung von Befunden in und aus Fremdsprachen. Auch hier werden Verständlichkeit und Genauigkeit verbessert, wenn das verwendete Vokabular genau definiert ist. Darüber hinaus profitieren (insbesondere auch multizentrische) Studien von eindeutig definierten Begriffen, da die untersuchten Phänomene so leichter aufzufinden und zu vergleichen sind.

Ein entscheidender Vorteil der strukturierten Befundung kann besonders den Jungradiologen helfen: Am Anfang einer radiologischen Karriere erscheint die Vielzahl an Fragestellungen und Untersuchungen zuweilen überwältigend. Ein vorgegebenes Schema in Form von strukturierten Befunden oder Checklisten hilft, sich auf Wesentliches zu konzentrieren und eine zeiteffektive Befundung neuer Fragestellungen zu erreichen, ohne zentrale Inhalte zu vergessen – auch wenn interdisziplinäre Erfahrung, beispielsweise aus Tumorboards, noch fehlt [8].

Warum also haben sich strukturierte Befunde (der zweiten und dritten Stufe) nicht schon längst flächendeckend durchgesetzt? Ein Teil der Antwort ist, dass sich diese Art der Befundung nicht auf jede Fragestellung gleich gut anwenden lässt. Generell gilt: Je gleichförmiger Untersuchungsarten und deren zu erwartende *Findings* sind, desto eher eignet sich ein vorgegebener, strukturierter Befundtext. Bei mehreren Fragestellungen oder multiplen erwarteten *Findings* kann das blinde Befolgen einer Befundmatrix viel Zeit in Anspruch nehmen, mehr Verwirrung stiften als Klarheit bringen und wesentliche Befundanteile untergehen lassen. Auch sind strukturierte Befundvorlagen nicht in allen Bereichen der Radiologie gleich gut ausgearbeitet und mangelnde Benutzerfreundlichkeit macht die mögliche Zeitersparnis noch oft zunichte.

In Bereichen mit hoher Akzeptanz strukturierter Befunde orientieren sich diese oft an Klassifikationen, die radiologische *Findings* gruppieren und mit klinisch-therapeutischen Konsequenzen verbinden. Insbesondere bei *Screening*-Untersuchungen haben sich diese etabliert. So können auch große Befundzahlen bewältigt werden, insbesondere, da in diesem Bereich eine hohe Anzahl unauffälliger Befunde oder Befunde ohne Notwenigkeit einer klinisch-therapeutischen Konsequenz anfällt. So haben sich Klassifikationen, wie beispielsweise BI-RADS, LI-RADS, LU-RADS und PI-RADS bereits überwiegend etabliert. Auch in der onkologischen Bildgebung nimmt die Anzahl strukturierter Befunde zu. Das *Staging* nach der TNM-Klassifikation und den RECIST-Kriterien wird zunehmend von den zuweisenden Kollegen gefordert. Vorteile von standardisiertem Vokabular, einheitlichen Klassifikationen und strukturierten Befundvorgaben zeigten sich auch in der Corona-Pandemie. Hierbei nimmt die radiologische Bildgebung eine wichtige Rolle in der Diagnostik ein. Die Entwicklung der RSNA-Klassifikation ([9]; Einteilung in „typisch“, „intermediär“, „atypisch“ und „negativ“ in Bezug auf COVID-19) oder der CO-RADS-Klassifikation [10] bietet nicht nur eine gute Übersicht und schnelle Erlernbarkeit der Erscheinungsformen einer pulmonalen COVID-19-Manifestation. Sie ermöglicht auch eine rasche Einschätzung der (prävalenzabhängigen) Erkrankungswahrscheinlichkeiten.

Neben kostenpflichtigen Angeboten stellen auch radiologische Gesellschaften und Arbeitsgruppen vermehrt strukturierte Befundvorlagen zur Verfügung. Darüber hinaus bieten einige von ihnen auch Implementierungssoftware an. Zudem werden sowohl international als auch in den einzelnen Sprachräumen große Anstrengungen unternommen, um ein einheitliches und eindeutiges Vokabular in der Radiologie zu finden. Eine Auswahl von Quellen zu strukturierter Befundung und definiertem Vokabular zeigt Tab. 1.

**Table 1:** 

Strukturierte Befundvorlagen^a^	
DRG	https://www.befundung.drg.de/de-DE/3199/befundvorlagen/
RSNA	https://radreport.org/home
Screening – Reporting and Data Systems	
ACR	https://www.acr.org/Clinical-Resources/Reporting-and-Data-Systems
Radiology assistant	https://radiologyassistant.nl
Vokabularien:	
RSNA/DRG	http://radlex.org
DRG	Glossar thoraxradiologischer Begriffe [11]
Fleischner Society	Glossary of terms for thoracic imaging [12]

^a^aktuell kostenlos zugänglich

## Fazit

Die strukturierte Befundung stellt gerade für junge Radiologen und bei rasantem medizinischem Fortschritt einen hilfreichen Leitfaden zu einem vollständigen und eindeutigen Befund dar. Hierbei können neben komplett vorgefertigten Befunden auch Checklisten, ein definiertes Fachvokabular und Textbausteine sinnvoll sein. Trotz einer Vielzahl einzelner Befundvorlagen (Tab. 1) ist jedoch nicht jede Fragestellung gleich gut für einen höheren Grad an Standardisierung geeignet. Gerade in Zeiten zunehmender Informationsmengen und Arbeitsverdichtung im medizinischen Sektor ist ein größeres Augenmerk auf benutzerfreundliche und zeiteffektive Anwendbarkeit der strukturierten Befundung nötig.
